# Nature’s Cardioprotective Sweetness: A Review of Dates as Functional Food in Hypertension

**DOI:** 10.3390/foods14244208

**Published:** 2025-12-08

**Authors:** Edwin Leopold Jim, Edmond Leonard Jim, Achmad Wildan, Antonello Santini, Fahrul Nurkolis

**Affiliations:** 1 Department of Internal Medicine, Royal Taruma Hospital, Jakarta 11470, Indonesia; edwinjim1@gmail.com; 2 Department of Cardiology and Vascular Medicine, Faculty of Medicine, Sam Ratulangi University, Manado 95115, Indonesia; edmondleonardjim@unsrat.ac.id; 3 Department of Agriculture Technology, Universitas Jenderal Soedirman, Banyumas 53122, Indonesia; achmad.wildan@unsoed.ac.id; 4 Department of Pharmacy, University of Napoli Federico II, Via Domenico Montesano, 49-8013 Napoli, Italy; 5 Master of Basic Medical Science, Faculty of Medicine, Universitas Airlangga, Surabaya 60131, Indonesia; fahrul.nurkolis.mail@gmail.com; 6 Medical Research Center of Indonesia, Surabaya 60281, Indonesia; 7 Institute for Research and Community Service, State Islamic University of Sunan Kalijaga (UIN Sunan Kalijaga), Yogyakarta 55281, Indonesia

**Keywords:** dates, *Phoenix dactylifera*, functional food, hypertension, cardiovascular health, polyphenols, antioxidant, anti-inflammatory, blood pressure, clinical trials

## Abstract

Dates contain numerous beneficial nutrients and bioactive compounds, including potassium, magnesium, dietary fiber, polyphenols, flavonoids, and essential fatty acids, which contribute to their antihypertensive effects. Mechanistically, these bioactives reduce oxidative stress, lower inflammation, enhance endothelial function through increased nitric oxide bioavailability, and modulate the renin–angiotensin system. Clinical studies demonstrate that regular, moderate consumption of dates can reduce systolic and diastolic blood pressure, improve lipid profiles, and positively regulate inflammatory and oxidative biomarkers without adverse metabolic or glycemic outcomes. Despite promising findings, variability in date cultivars, ripening stages, and limited standardized human trials necessitate further research. Larger, randomized clinical studies across diverse demographics are recommended to establish optimal dosage, confirm mechanisms of action, and assess long-term safety and effectiveness. This review investigates the potential of dates (*Phoenix dactylifera*) as a functional food for controlling blood pressure and protecting cardiovascular health in hypertensive individuals.

## 1. Introduction

### 1.1. Background

Hypertension remains a major global health concern and is widely recognized as a principal risk factor for cardiovascular disease, heart failure, stroke, and renal dysfunction [1,2]. Affecting nearly one-third of adults worldwide, it continues to be a predominant cause of morbidity and mortality due to cardiovascular complications [3]. As a result, public health strategies increasingly emphasize the role of diet and lifestyle interventions to complement pharmacological therapy. Within this context, functional foods have gained significant scientific interest for their ability to provide physiological benefits beyond basic nutrition.

Among these functional foods, dates (*Phoenix dactylifera*) have received growing attention due to their rich nutrient profile and long history of traditional use in many regions. Dates are naturally high in potassium and relatively low in sodium, a mineral pattern recognized as favorable for blood pressure regulation. They also contain substantial levels of polyphenols, flavonoids, dietary fiber, vitamins, and trace minerals, contributing to a biochemical profile associated with antioxidant, anti-inflammatory, and vascular-modulating properties [4,5]. Beyond their potential influence on blood pressure, dates are also associated with a range of other bioactivities reported across nutritional and biomedical research. These include antioxidant and anti-inflammatory actions, support for glycemic control, modulation of lipid metabolism, and protective effects observed in gastrointestinal, hepatic, and neurocognitive contexts. While these functions are not the primary focus of this review, they provide useful context for understanding the broader physiological relevance of *Phoenix dactylifera* as a functional food.

Although several studies have suggested beneficial effects, the current evidence from human clinical trials remains limited. Small-scale interventions indicate that date consumption may modestly reduce systolic and diastolic blood pressure, improve lipid profiles, and enhance markers related to oxidative balance and inflammation [6,7]. Some clinical trials on human volunteers show that date consumption could lower blood pressure and ameliorate related health outcomes [8]. Preclinical studies—including both animal models and in vitro experiments—support these findings by demonstrating mechanisms involving improved endothelial nitric oxide availability, reduced oxidative stress, inhibition of inflammatory cytokines, and protection against lipid peroxidation [7,9]. These mechanistic insights suggest that dates may influence multiple pathways relevant to cardiovascular health [7,9].

Despite these promising results, considerable gaps remain in the scientific literature. Variability among date cultivars, differences in ripening stages, and a lack of standardized dosing protocols across studies make it challenging to determine the consistency and generalizability of antihypertensive effects. Moreover, the small number of controlled human trials limits the ability to establish clear clinical recommendations. As such, further research is needed to confirm the therapeutic potential of dates, identify optimal consumption patterns, and evaluate their long-term safety and efficacy across diverse hypertensive populations.

### 1.2. Method

The research question in this study is how dates (*Phoenix dactylifera*) can be used to control blood pressure and cardiovascular protection in hypertensive patients. The objectives are to explore the nutritional and phytochemical properties of dates concerning cardiovascular health, to explore the molecular mechanisms of antihypertensive effects of dates, to analyze animal and human studies to evaluate the efficacy and safety of date consumption for blood pressure control, to compare dates with other functional foods that have been shown to affect blood pressure, and to consider issues related to the inclusion of dates in the diet of people who have hypertension.

The method used is a narrative review of the peer-reviewed nutrition literature and relevant biochemical and physiological investigations to determine the effects of date on blood pressure control. In addition, in vitro research is included to review the evidence for the mechanisms of date consumption in animal models. The efficacy of clinical trials is examined to assess the effects and safety of date as a functional food to reduce blood pressure in humans. Comparative reviews are also evaluated in the functional food domain.

A review was conducted using peer-reviewed nutrition literature and biochemical, physiological, and clinical databases (data retrieved from January 2017 till September 2025) Key databases including PubMed, Scopus, Web of Science, and Google Scholar were searched using terms such as “dates,” “*Phoenix dactylifera*,” “hypertension,” “blood pressure,” “cardiovascular health,” “functional food,” “polyphenols,” “antioxidants,” and “clinical trials.” Inclusion criteria encompassed articles focusing on nutritional and phytochemical properties, mechanisms of antihypertensive action, animal and human efficacy studies, comparative analysis with other functional foods, and safety data related to hypertensive populations. Studies were selected based on relevance, rigor, and recency to ensure comprehensive and accurate review findings.

## 2. Botanical and Nutritional Profile of Dates

**Figure 1:**
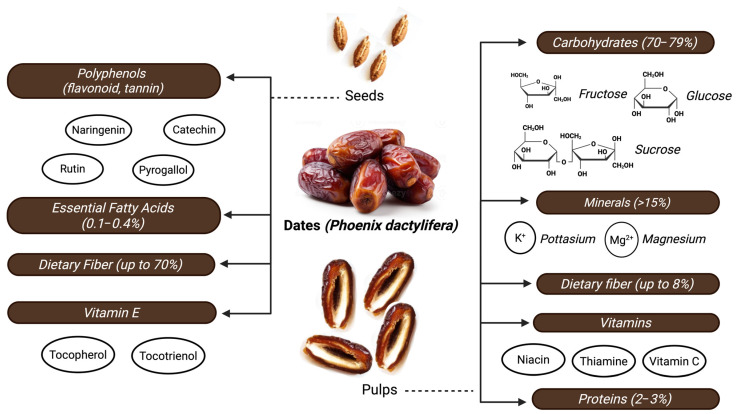
Schematic representation of the botanical structure of *Phoenix dactylifera* fruit showing major nutrients and bioactive compounds relevant to blood pressure regulation. Created by the authors using BioRender.com (accessed on 21 August 2025).

To contextualize the blood-pressure-lowering potential of dates, this section provides a concise overview of their chemical constituents and nutritional profile. Highlighting key phytochemicals, minerals, and dietary fibers offers a foundation for understanding the mechanisms through which dates may influence vascular function and contribute to hypertension management (Figure 1).

### 2.1. Phytochemical Composition

The carbohydrate composition of dates is mainly simple sugars: fructose, glucose, and sucrose. Their ratio by weight can vary from 70 to 79% [10]. Dates are an energy-dense fruit that provides a relatively high amount of calories (up to 282 calories/100 g) and may not be the most suitable option for populations at risk of developing metabolic syndrome. Studies have demonstrated that some varieties of dates can be included in the diet without causing great fluctuations in glycemic parameters; therefore, it can be inferred that their consumption is appropriate in therapeutic dietary interventions for hypertensive patients [10,11,12,13]. In addition to their carbohydrate composition, the protein content in dates (2–3% approximately) makes them a source of essential amino acids such as lysine, leucine, valine, and threonine [11]. The fat content in the flesh of dates is low (0.1–0.4%); however, it is present in high amounts in their seeds. Among the fats found in dates, certain essential fatty acids with anti-inflammatory activity can be found, which is a remarkable characteristic for fruits with high sugar content [10,13].

The mineral composition of dates, particularly their relatively high levels of potassium and magnesium, may contribute to their potential relevance for cardiovascular health [14]. Both minerals are well known for their vasodilating properties, which improve fluid balance and blood pressure regulation. 100 g of dates may represent over 15% of the daily recommended intake (DRI) for both potassium and magnesium [10,11,13]. Furthermore, dates present other trace elements, namely iron, copper, selenium, and zinc, which may have a significant effect on vascular health. Selenium and zinc enhance antioxidant capacity and also participate as cofactors for several enzymes, which play an important role in cardiovascular health [10]. The presence of peculiar trace minerals like boron, fluorine, and manganese makes dates a novel functional food [11], distinguishing it from other sweet fruits, which are not usually recommended in diets of hypertensive patients.

Dietary fiber can be found in high concentrations in dates, its composition varying significantly depending on the date seed and its flesh [13,15]. Date flesh contains approximately 8% of fiber, while the seeds can reach up to 70% of fiber [13,15]. Dietary fiber in dates consists mainly of insoluble components and contributes to the proper functioning of the gastrointestinal tract. The regular consumption of dietary fiber enhances stool bolus size and prevents constipation and related colon disorders [15]. The benefits of dietary fiber are also associated with an improvement in metabolic parameters. It is suggested that it helps reduce blood sugar fluctuation after ingestion of foods, which may enhance glucose control. This parameter should be taken into consideration in patients who have hypertension, as it is recommended to maintain stable blood sugar levels [13]. Furthermore, the composition of dietary fiber affects the absorption of cholesterol. The intake of dietary fiber may reduce the absorption of cholesterol in the small intestine, and subsequently, cholesterol levels may decrease, as well as LDL concentrations [16]. In summary, the consumption of dietary fiber in dates has multiple direct and indirect effects on hypertension. Dietary fiber may exert positive effects directly on vascular function and also enhance several metabolic processes that contribute to reduced cardiovascular risk in hypertensive patients [10].

Phenolic composition in dates is complex, with some varieties showing concentrations of 1000 mg/kg of total phenolics in pyrogallol equivalents or tannic acid equivalents [10]. Dates present high flavonoid concentrations, especially in the seeds. These flavonoids consist mainly of naringenin and rutin [10,15]. Phenolic compounds in date seeds enhance antioxidant activities through several mechanisms. They are known to scavenge free radicals such as 2,2-diphenyl-1-picrylhydrazyl (DPPH) and Superoxide Anion Radical Scavenging Activity (SARSA) [15], and are considered to be suitable candidates to prevent or alleviate oxidative stress induced by hypertension [17]. Some flavonoids in dates, namely catechic tannins and pyrogallol derivatives, possess stronger antioxidant activities compared to widely commercialized antioxidants [15]. The content of phenolic compounds varies according to date varieties; for instance, Halawi dates contain higher levels of phenolic compounds than Medjool dates [10]. The maturation stages also have an influence on the amount of phenolic compounds. Studies have demonstrated that Khalal maturation stages may show higher antioxidant activity than Tamr stages [11]. Phenolics may have a significant influence in the prevention and alleviation of hypertension through their ability to reduce endothelial dysfunction and enhance antioxidant processes [10].

Dates are known to be sources of several vitamins, such as niacin, thiamin (B-complex), and ascorbic acid, all of which can be found in the fruit flesh, as well as analogs of vitamin E [10]. On the other hand, significant amounts of vitamin E, primarily tocopherols and tocotrienols, are mainly found in the seeds. Some date seed varieties can provide up to 34 mg/100 g α-tocotrienol [13]. Vitamins are known for their influence on several physiological processes that benefit hypertension, with tocopherols being known for their strong antioxidant abilities and B-complex vitamins enhancing antioxidant defenses [10]. Vitamin E has been shown to be helpful in reducing endothelial dysfunction and improving antioxidant enzyme activities [13].

Overall, the variations in phytochemical composition, even within the same variety, make the therapeutic benefits of date consumption uncertain. Specific varieties of dates richer in phenolic compounds, like the Halawi, and harvesting fruits that have higher antioxidant activity may be useful to reduce oxidative stress and the development of hypertension. The concentrations of phenolics, flavonoids, minerals, and other bioactives in *Phoenix dactylifera* are influenced by a suite of agronomic and environmental (edaphoclimatic) factors, including cultivar genetics, soil type, irrigation regime, local climate, and fertilizer practices. Comparative studies and reviews have demonstrated that geographical origin and orchard management produce measurable differences in total phenolic content, mineral profiles, and antioxidant capacity across date samples, such that cultivar-specific nutritional claims should be interpreted in the context of provenance and agronomic conditions [18]. Ripening stage, post-harvest processing, and analytical method effects further modulate the detectable levels of bioactives. Multiple reports show that phenolic fractions and tannins change markedly during ripening (e.g., kimri → khalal → rutab → tamr), with wide varieties exhibiting the highest tannin/phenolic levels at intermediate stages and a decline as sugars increase toward full maturity [19,20]. Processing (drying, blanching, roasting) and storage may either concentrate or degrade specific compounds depending on conditions (temperature, time, light), and different extraction protocols (solvent polarity, temperature, sonication/ultrasound, pressurized liquid extraction) produce substantially different yields and profiles of phenolics and flavonoids. Thus, apparent discrepancies among studies often reflect differences in ripeness, post-harvest handling, analytical extraction technique, and assay choice rather than the absolute absence/presence of a given compound. However, more standardized profiling and comparative analyses are needed to better understand the antihypertensive activity of date consumption.

### 2.2. Bioactive Components

Date seeds and pulp are sources rich in bioactive compounds, which may contribute to their cardiovascular effects (Table 1). Seeds can have up to 71.5–88.9 g/100 g of dietary fibers and up to 12.13 g gallic acid equivalents (GAE)/100 g of total phenolic content, with Sukkari dates having a more favorable concentration of functional compounds [21]. Consuming both pulp and seeds can target different cholesterol-reducing and LDL-C-lowering pathways, which may affect the inflammatory markers, highlighting the need for the inclusion of whole fruits in order to improve their cardiovascular health benefits (Table 1).

Both the pulp and seeds have distinct but overlapping biochemical profiles, which may suggest potential for tailoring supplements for at-risk groups such as individuals with hypertension or metabolic syndrome. Date seeds contain vasoprotective and antioxidant catechic tannins, naringenin, and rutin [21]. Since the antioxidant capacity of date seeds can be compared with that of butylated hydroxytoluene (BHT) (a synthetic antioxidant), they could be incorporated in food nutraceutical supplements to confer cardioprotective effects and to valorize waste products from agricultural practices [15].

However, the concentrations of bioactive constituents are highly variable for different cultivars and depend also on their degree of ripening and the process they undergo. For instance, unripe products made of intermediate Medjool and Confitera varieties contained concentrations up to 1.4 g GAE/100 g and 874 mg retinol equivalents (RE)/100 g for total phenolics and flavonoids, respectively [22]. Blanching also significantly increases the antioxidant capacity of certain date cultivars. This emphasizes that both the appropriate selection of date cultivars and post-harvest treatments are important for enhancing the blood pressure-lowering effect of the date.

Dates also contain functional carbohydrates, such as β-glucan, cellulose, and fructans. These functional carbohydrates improve gut health and exert anti-inflammatory, antioxidative, and lipid-lowering effects, which may play a role in blood pressure control [23]. They can influence various metabolic pathways relevant to the prevention of cardiovascular disease. Therefore, it may be more relevant to consume dates as a whole fruit and not the isolated components, to prevent hypertension [24]. Dietary fibers can also interact with polyphenols present in dates, such as chlorogenic acid, cinnamic acids, and flavonoids, leading to further investigation on the effects of the co-presence of these compounds in the vasculature of hypertensive individuals [25].

As indicated above, many date-derived bioactive compounds, such as dietary fibers and polyphenols, were seen to reduce oxidative stress and improve the lipid profile of an organism in vitro and in vivo [26,27]. They have also been shown to prevent the development of key cardiovascular diseases and complications of hypertension by directly impacting vascular risk factors like dyslipidemia and endothelial dysfunction [28,29]. Since they also possess antioxidative effects, their addition as a functional food to the general guidelines on hypertension management would be adequate to ensure cardiovascular protection. In relation to dates, research on plant bioactives has suggested a preventive role against cardiovascular diseases, thus endorsing the intake of this food in the diet of people with hypertension [30].

Tannins, flavonoids, and carotenoids in dates act on mechanisms involved in inflammation and atherogenesis. Studies on human vascular smooth muscle cells treated with pro-inflammatory factors revealed that tannins, flavonoids, and carotenoids act on different cell signaling pathways and regulate genes involved in the inflammatory response [31]. This confirms that such polyphenolic and carotenoid compounds may exert protective effects in inflammatory or oxidative stress conditions in hypertension [32].

**Table 1:** Summary of key nutritional and phytochemical components of dates (*Phoenix dactylifera*) and their proposed antihypertensive mechanisms.

Component Category	Specific Constituents	Representative Concentrations (Approx.)	Primary Physiological Functions	Antihypertensive Mechanisms	Key References
Minerals	Potassium, Magnesium, Iron, Copper, Selenium, Zinc, Manganese, Boron	K ≥ 600 mg/100 g; Mg~50–60 mg/100 g	Electrolyte balance; vascular smooth muscle relaxation; antioxidant enzyme cofactors	Natriuresis; improved arterial compliance; enhanced NO synthesis; reduced oxidative stress	[10,11,13,14]
Dietary Fiber	Insoluble and soluble fiber; β-glucan; fructans; cellulose	Flesh~8%; Seeds up to 70%	Improves glycemic control; enhances satiety; modulates lipid absorption	LDL-C reduction; stable postprandial glucose; improved metabolic function	[10,13,15,16,20,21]
Phenolic Compounds and Flavonoids	Naringenin, Rutin, Catechic tannins, Pyrogallol derivatives, Phenolic acids	Up to 1000 mg/kg; seeds richer	Antioxidant and anti-inflammatory activity	Scavenges ROS; inhibits lipid peroxidation; enhances eNOS activation; reduces IL-6 and TNF-α	[10,11,15,18,19,28]
Vitamins	Vit B-complex, Vit C, Vit E (α-tocotrienol, tocopherols)	Seeds up to 34 mg/100 g α-tocotrienol	Redox regulation; metabolic function	Boosts antioxidant enzymes (SOD, catalase, GPx); protects endothelium	[10,11,13,31,32]
Sugars (Carbohydrates)	Fructose, Glucose, Sucrose	70–79%	Energy supply; moderate GI response	Prevents acute glycemic spikes; stabilizes vascular tone	[10,12,13]
Fatty Acids	Linoleic acid, Oleic acid (mainly in seeds)	Seeds show a higher fat fraction	Anti-inflammatory activity	Reduces vascular inflammation; supports membrane and microvascular stability	[10,13,18]
Seed Bioactives	High phenolic content; tannins; flavonoid-rich extract	TPC up to 12,128 mg GAE/100 g	Antioxidant and LDL-protective profile	ACE inhibition; reduced oxidized LDL; improved endothelial reactivity	[15,18]

### 2.3. Date Seeds—Processing, Availability, and Consumption

Date seeds are an abundant agro-industrial by-product that can be converted into several value-added ingredients (seed flours, defatted seed meal, cold-pressed or solvent-extracted oils, polyphenolic extracts, and roasted “date-seed coffee” alternatives) through relatively simple processing steps. Typical processing begins with cleaning and drying of whole seeds, followed by dehulling (when necessary), roasting or blanching to improve flavor and reduce microbial load, and fine milling to produce seed flour or powder suitable for food formulations. For oil or lipophilic antioxidant recovery, conventional solvent extraction and Soxhlet systems have been widely reported, while greener approaches (ultrasound-assisted, hydrothermal pretreatment, pH-shift, and subcritical techniques) are being optimized to increase yield and preserve thermo-sensitive bioactives [13,31]. To protect phenolics during manufacturing and digestion, encapsulation techniques (liposomes and spray-drying) have been successfully trialed, enabling incorporation of seed extracts into dairy matrices (e.g., yogurts) and bakery products with improved bioaccessibility [6,28]. These processing pathways are described in recent valorization reviews and method papers that demonstrate feasible scaling from lab to industry for date-producing regions and beyond.

In practice, date seeds are increasingly available in consumer and industrial forms: as date-seed flour (used as partial wheat flour replacer or fiber-rich ingredient in biscuits and breads), defatted seed meal (protein/fiber concentrate), seed oil (nutraceutical, cosmetic, and culinary uses), polyphenolic extracts (nutraceutical ingredients), and roasted seed beverage (date-seed coffee) (a palatable antioxidant-rich drink) [25]. Food-form studies report that seed flour inclusion levels of ~10–20% in bakery formulations retain acceptable organoleptic properties while delivering added fiber and antioxidant capacity, and consumer testing on date-seed products has shown favorable acceptance in date-producing countries (Middle East, North Africa) where seeds are a low-cost by-product [17]. For consumption recommendations, minimally processed seed flour (heat-treated and milled) or encapsulated seed extracts offer pragmatic routes to deliver polyphenols and fiber in a food matrix; defatted seed meals and oils can be used in specialized formulations but require attention to extraction solvent residues and oxidation stability. Safety considerations include ensuring hygienic drying, appropriate heat treatments to reduce microbial or anti-nutritional concerns, and clear labeling for potassium/energy content when targeting populations with CKD or diabetes. Overall, date seeds represent a scalable, culturally acceptable ingredient platform that can be integrated into staple foods or developed as specialty nutraceuticals pending standardized processing and regulatory evaluation.

## 3. Mechanisms of Blood Pressure Regulation

**Figure 2:**
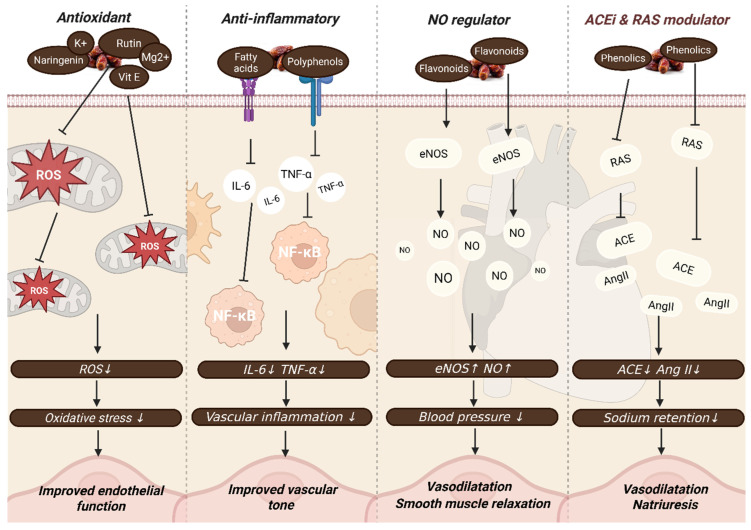
Proposed schematic of how date-derived nutrients and phytochemicals exert antihypertensive effects via antioxidant, anti-inflammatory, endothelial modulation, and ACE-inhibitory pathways. Created by the authors using BioRender.com (accessed on 21 August 2025).

Comprehension of the complex biological systems by which dates have an impact on blood pressure is critical for understanding the mechanisms that support their therapeutic efficacy (Figure 2). This section explores the role of dates in influencing cardiovascular health through their antioxidant, anti-inflammatory, and vascular modulation properties. These features contribute to the quest for functional foods that may be included in dietary strategies for managing high blood pressure.

### 3.1. Antioxidant and Anti-Inflammatory Effects

**Figure 3:**
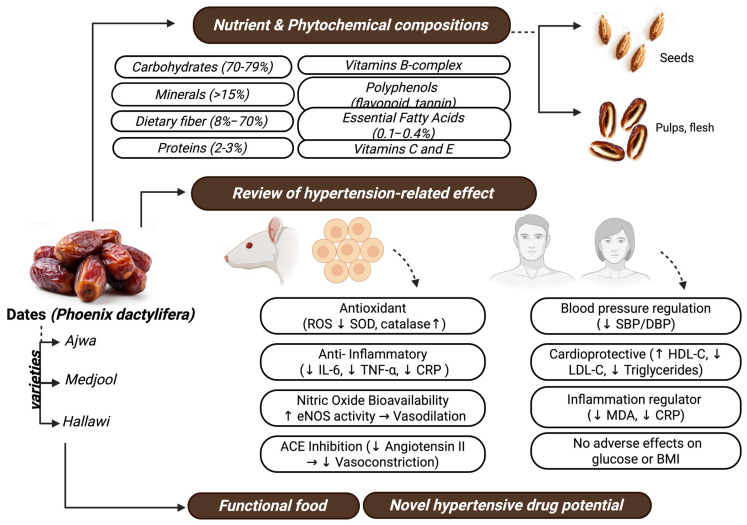
Proposed mechanistic framework illustrating how nutrient and phytochemical compositions of date fruit (*Phoenix dactylifera*) contribute to blood pressure regulation. Created by the authors using BioRender.com (accessed on 21 August 2025).

Dates have been confirmed for their strong antioxidant and anti-inflammatory capabilities, making them an ideal functional food for cardiovascular health. They are made up of several phenolic compounds and flavonoids found in both the fruit flesh and the seeds, and are capable of preventing free radical reactions (Figure 3). These bioactives block lipid peroxidation, therefore minimizing oxidative stress in vascular tissues and lowering the chances of hypertension [33]. Research utilizing DPPH and 2,2′-azino-bis(3-ethylbenzothiazoline-6-sulfonic acid) (ABTS) radical scavenging tests shows how dates’ free radical scavenging activities effectively reduce reactive oxygen species in hypertension. It is also suggested that polyphenol extracts from the pulp and seeds of the Khalas and Sukkari date cultivars significantly scavenged free radicals and enhanced antioxidant enzyme activity in hypercholesterolemic rats [21]. These facts help justify the hypothesis of using dates for the treatment of vascular oxidative stress. Further studies are recommended in order to better understand the interaction of date antioxidants with the endogenous enzymatic antioxidant defenses.

Dates contain carbohydrates, minerals (particularly potassium and magnesium), vitamins (B-complex, C, and E), polyphenols, essential fatty acids, and dietary fiber. These bioactives exert antioxidant (↓ ROS, ↑ SOD, ↑ catalase), anti-inflammatory (↓ IL-6, ↓ TNF-α, ↓ CRP), and endothelial-modulating effects (↑ nitric oxide bioavailability via eNOS activation). They also inhibit angiotensin-converting enzyme (ACE), thereby reducing angiotensin II-mediated vasoconstriction. Collectively, these mechanisms contribute to improved blood pressure regulation, cardioprotection, and overall vascular health without adverse effects on glucose metabolism or BMI, highlighting the potential of dates as both a functional food and a source for novel antihypertensive agents. Also, dates have been linked to the restoration of antioxidant enzymes, such as catalase, superoxide dismutase, and glutathione peroxidase [34,35]. These enzymes play an important role in controlling reactive oxygen species levels by scavenging free radicals, maintaining cellular redox, and defending against endothelial damage. The polyphenol extract of Ajwa dates reduced oxidative stress and improved antioxidant enzyme activity in hypercholesterolemic rats [36]. Also, there is evidence that Ajwa dates were capable of restoring the endogenous antioxidant enzymes and stabilizing oxidative stress damage in myocardial models [37]. Together, these studies suggest the potential for dates to maintain cardiovascular health by reducing oxidative stress caused by hypertension. However, the lack of human intervention trials requires further investigation regarding date consumption in differing health and diet conditions and its influence on antioxidant enzymes.

Aside from their antioxidant activities, date bioactives are able to reduce pro-inflammatory cytokines, such as interleukin-6 (IL-6) and tumor necrosis factor-alpha (TNF-α). In another research, the consumption of polyphenol-rich Ajwa dates had a suppressing effect on inflammation as well as myocardial apoptosis in rodents with cardiomyopathy [38]. Additionally, histological research showed improved myocardial architecture after date supplementation, suggesting improved cellular health and overall functional efficacy. This is further supported, where evidence showing decreased levels of inflammation markers, such as C-reactive protein (CRP), when consuming date fractions [21]. Thus, it can be suggested that dates have the capacity to combat inflammation in hypertension in part by modulating the aforementioned biochemical inflammatory markers. However, more research should be performed in order to test this hypothesis. Furthermore, it is recommended to confirm the dose responsiveness of date supplementation in the general population.

One key method that dates use to protect from cardiovascular harm is through the prevention of low-density lipoprotein (LDL) oxidation [39]. Aside from their antioxidant effect on free radicals, polyphenols, such as flavonoids in dates, are able to improve endothelial nitric oxide synthase (eNOS) activity, resulting in the increased availability of nitric oxide, which promotes vascular health. Additionally, phenolic acids, flavonoids, and carotenoids, which are abundant in dates, have an LDL protective property while also contributing to vascular function and vasodilation [40]. Also, LDL-C and oxidized LDL were reduced after supplementation with polyphenol extract of Ajwa dates in hypercholesterolemic animal models [36]. The combined results of these experiments demonstrate the protective effect that date compounds have on the development of atherosclerosis via the mechanism of LDL protection. The fact that oxidative stress increases the likelihood of hypertension suggests that dates should reduce blood pressure, partially through mechanisms involving LDL protection, and should be further researched as a functional food that can reduce hypertension in susceptible individuals.

In comparison, the antioxidant and anti-inflammatory mechanism of action for dates in comparison with other plant functional foods is an intriguing topic to discuss. Evidence states that plant phytochemicals are key agents for fighting oxidative stress and inflammation, both of which play several roles in disease etiology [41]. Ajwa dates are similar, possessing high amounts of flavonoids and polyphenols comparable to other functional foods, such as green tea and berries. Pleiotropic benefits of date extracts in combating oxidative stress in cardiomyopathy [38]. Similar results of date extracts preventing oxidative damage have been shown by [37]. However, few studies have focused on the broader bioactivities that can be considered when comparing dates with other plant functional foods, making it difficult to construct a hierarchy for functional foods to treat conditions, such as hypertension.

The use of dates for the practical application of preventing or reversing hypertension is supported by animal and human studies. Research showed lower total cholesterol, LDL-C, and CRP, as well as elevated catalase and glutathione peroxidase in the hypercholesterolemic model [21]. Also, improvements in aortic blood flow and inflammatory markers were observed in rodents supplemented with Ajwa dates compared to controls in the study performed by [38]. Overall, these data support the functional effectiveness of dates in regard to the reduction in blood pressure in experimental models. Dates are beneficial in treating hypertension and type 2 diabetes due to their bioactives having antioxidant and anti-inflammatory activities [40]. Additional research is recommended in order to identify practical methods of date consumption to reduce the likelihood of hypertension.

In conclusion, the mechanism of action of date bioactives is based on antioxidant and anti-inflammatory properties. While the functional efficacy of dates in treating hypertension is based upon only a handful of studies, these studies offer convincing evidence of their value. Thus, date consumption may prevent or reduce hypertension through the means of improved inflammatory and oxidant pathways. Further research should be conducted in the arena of the mechanistic properties of date consumption for hypertension.

### 3.2. Vascular Function Modulation

Date consumption has potential as a dietary intervention to improve vascular function in hypertension. The high polyphenol and flavonoid content of dates is linked to enhanced endothelial function by promoting increased nitric oxide (NO) bioavailability, which is crucial for vasodilation. Clinical studies have shown improvements in vascular elasticity following date consumption [7,37]. These improvements may be due to bioactive compounds stabilizing NO synthase activity, alleviating endothelial dysfunction, and supporting vasodilation. However, further studies are required to ensure these findings are reproducible across diverse populations.

Inhibition of NO oxidative degradation and promotion of eNOS gene expression by date bioactives may represent new mechanisms to mitigate endothelial dysfunction. These mechanisms are especially important for hypertensive individuals, as impaired endothelium-dependent vasodilation is prevalent in this group. Phenolic-rich date consumption improves endothelial health by enhancing certain endothelial biomarkers in hypertensive individuals [7]. This suggests that careful selection of date cultivars (i.e., choosing date varieties richer in phenolic compounds) could significantly optimize health outcomes. Hallawi and Ajwa date cultivars have been shown to contain relatively high concentrations of phenolic compounds [7]. Clinical studies on human health using specific date extracts may be necessary to corroborate the specific molecular mechanisms involved and to ensure sustained benefit in the long term.

Dates improve vascular function by improving both microvascular and macrovascular reactivity through mechanisms elucidated in preclinical studies [7]. Additionally, the results of in vitro studies using isolated blood vessels showed that dates are capable of inducing arterial dilation through vascular endothelial-dependent relaxation. Dates were observed to have direct beneficial impacts on endothelial health in a hypertensive population [7]. These findings offer new evidence of their role in cardiovascular health strategies. Future randomized controlled trials should further validate these findings on vascular health in larger, more diverse hypertensive populations. Because endothelial dysfunction is a predictor of cardiovascular morbidity, the consumption of date-derived bioactives may aid in hypertension management by restoring endothelial health. Date bioactives modulate various endothelial pathways, supporting improved vasodilation and decreased systemic vascular resistance [37]. These effects may reduce the incidence and severity of hypertension complications. The effective daily dosage of date bioactives and the specific mechanisms behind their health effects remain to be further explored in clinical intervention studies.

High potassium and low sodium content are beneficial in the management of blood pressure. Potassium and sodium in the diet affect blood pressure through different mechanisms. Potassium intake lowers blood pressure through natriuresis, reduced vascular resistance, and improved arterial compliance. Studies mention the blood pressure-lowering properties of potassium [7,42]. The biological activities of potassium modulate the renin–angiotensin–aldosterone system, thereby decreasing vasoconstriction, fluid retention, and systemic blood pressure [43]. Furthermore, natriuresis increases the production of vasodilating factors in endothelial cells. Epidemiological and mineral balance studies support the role of potassium in regulating blood pressure. However, additional human intervention studies are needed to compare the hypotensive effect of dietary potassium in the form of dates versus other sources of dietary potassium. The nutritional benefits of potassium are important for people with impaired potassium homeostasis, such as those with metabolic syndrome or chronic kidney disease, and in populations with high-sodium diets. Dates are both a culturally acceptable and nutritionally effective food for managing high blood pressure [42]. Although potassium supplementation has known blood pressure-reducing effects, it remains to be demonstrated whether dates can significantly counteract the adverse effects of high sodium intake and impaired potassium handling.

In vitro studies have demonstrated that date extracts can significantly inhibit ACE and have potential applications in lowering blood pressure. Studies have reported the blood-pressure-lowering capabilities of flavonoids and isosorbide, bioactive compounds of dates, in relation to their ACE inhibitory activities [42,44]. Selected date extracts exhibit greater ACE inhibitory activities than currently marketed ACE inhibitor pharmaceuticals. These results allow for the possibility of a synergy between date-derived bioactives and pharmaceuticals to lower blood pressure in the management of hypertension. Certain extracts of dates were observed to show ACE inhibitory activity at 71% compared to 69% of captopril, the most effective ACE inhibitory drug [42]. This provides an opportunity to explore the ACE-inhibiting activity by varying parameters such as dosage and time duration to observe if it is more effective than captopril or other ACE inhibitors. This would aid in understanding the dose that is required to improve the health benefits of dates and how they interact with other drugs for better health results.

LDL cholesterol oxidation is also a critical component in enhancing vascular function. The antioxidant abilities of dates were mentioned, where they scavenge reactive oxygen species, inhibiting the oxidation of LDL cholesterol [37]. Phenolic acids and carotenoids stabilize eNOS activity, improving endothelial cell vascular reactivity, thereby ameliorating hypertension [45]. By inhibiting oxidative damage to vascular endothelial cells, date bioactives can protect vascular health, improve vascular function, and decrease blood pressure. Clinical and mechanistic human studies must confirm the extent to which the antioxidant function of date bioactives leads to measurable, practical dietary and health benefits.

The synergistic effects of polyphenols, potassium, and dietary fibers in date bioactives highlight their complex interplay on blood pressure regulation. Dates lower both mean and diastolic blood pressure, improve NO production, increase vasodilation, and reduce blood pressure variability [7]. These physiological benefits are not restricted to the effects of single nutrients but are a result of synergistic interactions with other nutritional components of dates, which lead to the modulation of metabolic parameters. To effectively utilize the health-promoting properties of dates to address hypertension, future research must aim to identify the most beneficial combination of date-derived bioactives and elucidate how they work together.

Compared to other plant-based functional foods such as probiotics, dates regulate blood pressure through a combination of nutritional factors and phytochemicals with mechanisms related to the induction of natriuresis and endothelial-dependent vasodilation, rather than regulating blood pressure through gut-microbiota-related mechanisms such as increased expression of ACE and renin genes as observed with probiotics [46]. While the effect of probiotics on blood pressure is mediated by regulating the gut microbiota, the effect of dates is mediated by minerals such as potassium and magnesium and compounds such as polyphenols [46]. The difference in the mechanism of blood pressure regulation leads to differences in the effectiveness of the blood-pressure-reducing effect when the blood pressure of a hypertensive patient is reduced. The regulation by the intestinal flora may not cause significant changes in the blood pressure of a hypertensive patient [47]. For minerals and polyphenols, the blood pressure is relatively reduced. These findings could lead to new treatment modalities for hypertension, such as combinations of different kinds of fermented foods with different date products.

Dates are a promising functional food for hypertension prevention and management because of their direct blood pressure-lowering capability, acting through both nutrient and phytochemical mechanisms. Date bioactives lower the elevated systolic and diastolic blood pressures in hypertensive individuals, thus reducing the burden on vascular health [42]. The high cultural acceptability of dates, combined with a well-established safety profile, supports their being part of a dietary therapy for hypertension. However, further mechanistic research on date bioactives in vivo is needed to confirm and determine optimal dosages and to tailor date-based intervention to individual clinical conditions. Regular date consumption is associated with improvements in vascular stiffness and lipid metabolism. Human intervention studies have shown that regular consumption of dates improves blood lipid profiles. In addition, improvements in endothelial function, systolic blood pressure, mean arterial pressure and diastolic blood pressure, pulse pressure, and blood lipid profiles after consumption of 100 g of dates were noted [7]. There are, however, gaps in information about the optimal form of dates, their effective daily dose, and their long-term effects and suitability among diverse demographic groups.

The safety of dates, even in metabolically challenged individuals, allows them to be effectively incorporated into a treatment strategy for managing hypertension. Studies that incorporate dates have had positive safety and tolerance outcomes even with certain parameters [42]. This confirms the ability of individuals to comfortably consume and adhere to a date-based diet strategy. In general, dates have a pleasant taste, acceptable mouthfeel, and do not affect the palatability of most foods. The positive effects of date consumption on endothelial function and blood pressure control are observed to be achieved without affecting renal or glycemic parameters, even in individuals who are diabetic or are being treated for chronic kidney disease [7]. Nevertheless, it is recommended that controlled trials be employed to address concerns about excessive sugar content and to demonstrate the beneficial effects of dates in diverse demographics, particularly in vulnerable populations such as diabetics and individuals with chronic kidney disease.

Consumption of dates not only provides health benefits beyond basic nutrition, but the bioactive components in dates also promote vascular health. Therefore, incorporation of dates into a culturally appropriate dietary regime benefits patients beyond reducing high blood pressure, by promoting better adherence to a recommended nutritional diet. Furthermore, these benefits occur without an increase in weight gain or alterations in blood lipids [7,42]. Future research should develop precision nutrition strategies using various bioactive compositions of dates to address the complications of hypertension. To complement qualitative descriptions of date bioactivity, Table 2 synthesizes quantitative findings from human and animal studies, highlighting dose ranges, intervention durations, and measurable outcomes such as blood pressure reduction, antioxidant enhancement, lipid regulation, and inflammatory modulation. The separation of human and animal data clarifies translational relevance while emphasizing dose–response variability across study designs.

## 4. Clinical Evidence and Safety

Regular date consumption, as a functional food, may be beneficial in the management of hypertension. According to intervention studies, the oral consumption of approximately 100 g of dates for at least four weeks is sufficient to reduce blood pressure in adults with hypertension [48]. Their polyphenols also inhibit oxidative stress and improve endothelial health [7,49]. The food matrix in dates slows potassium absorption, as it is a nutrient-dense whole food with fiber and polyphenols, whereas oral potassium supplementation has a fast absorption rate [49]. Although potassium is a natural antihypertensive element, the isolated forms may not have the same effect as the potassium sourced in dates. Also, different date varieties and processing methods may vary in their benefits. Further research will have to investigate this point.

Potassium, a prominent mineral in dates with concentrations often over 600 mg per 100 g, counters the effect of sodium on blood pressure. It has been known to increase sodium excretion and improve vascular resistance and arterial compliance for those with hypertension [7,49]. While potassium can be obtained as an oral supplement, the natural form found in dates provides a more holistic combination of bioactive components that leads to a more sustainable impact on blood pressure. An intervention study involving older participants who consumed varieties of dates, such as Ajwa, for six weeks displayed an average reduction of 14 mmHg in systolic blood pressure and 8.5 mmHg in diastolic blood pressure [50]. Although dates have demonstrated significant antihypertensive qualities, the dose, frequency, and intervention period may not be consistent to achieve a clinical effect.

**Figure 4:**
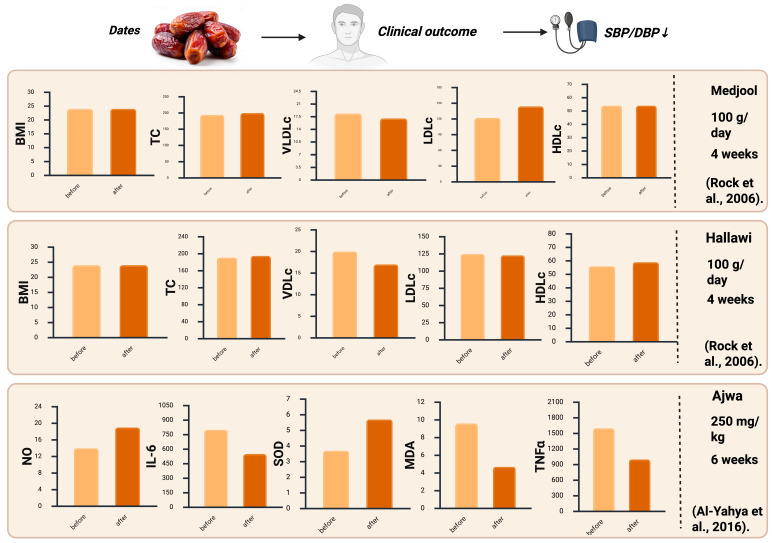
Magnitude of systolic and diastolic blood pressure reduction, and changes in key inflammatory and oxidative biomarkers, reported in human intervention trials of date consumption. Created by the authors using BioRender.com (accessed on 21 August 2025).

Intervention trials have reported that date consumption may have beneficial impacts on the lipid profiles of participants (Figure 4). Hypertension and dyslipidemia are strongly linked to cardiovascular risks [8,49]. Clinical intervention trials have shown that the regular consumption of dates over a four-week period, Hallawi or Medjool, improved the levels of triglycerides and total cholesterol, with no significant effect on blood glucose levels in the at-risk population [7,8,49]. Blood glucose levels need to be managed properly to avoid negative implications for patients’ health. Moreover, trials have demonstrated mild improvement of LDL cholesterol and HDL cholesterol, both of which contribute to a positive change in the atherogenic lipid profiles of hypertensive participants (Figure 3) [8,51]. Whether the improvement in lipid profiles is a result of the high content of fiber or polyphenols in dates, or both, will need to be assessed in future clinical intervention trials.

Several clinical intervention trials found that serum antioxidant capacity was enhanced and levels of MDA (malondialdehyde), a marker for lipid peroxidation, and indicators of oxidative damage, were reduced in those who regularly consumed dates [38,49]. The anti-inflammatory effect of dates has also been shown in multiple studies, as significant reductions in C-reactive protein (CRP) and tumor necrosis factor-alpha (TNF-α) were reported [49]. These observations suggest that the antihypertensive effect of date consumption is mediated at least in part by a reduction in oxidative and inflammatory stress [49]. Furthermore, date consumption has been shown to improve antioxidant enzyme activity, such as superoxide dismutase and catalase [38]. While these intervention studies have demonstrated that date consumption can lower hypertension, reduce oxidative and inflammatory stress, and improve blood lipid profiles, the ideal consumption amounts and duration required to maintain clinical efficacy need to be analyzed more carefully, ideally within clinical settings.

According to multiple human clinical intervention trials, date consumption did not impact body mass index in healthy adults or patients, and the interventions lasted from four to sixteen weeks [8,52]. Dates are a nutrient-dense, high-calorie food, but their ability to impact body mass index will determine their potential inclusion as an adjunct therapy. Their beneficial effect on the blood glucose control of hypertensive patients will also determine their viability as a snack among this population, who often struggle with body weight and metabolic disorders [8,49]. However, studies that included date consumption coupled with caloric control reported a positive impact on body weight and improved glucose metabolism of participants due to the high dietary fiber in dates, which induces early satiety [49]. Despite evidence of glycemic neutrality in several intervention studies, the naturally high sugar content of dates still warrants caution in hypertensive patients with comorbid diabetes, metabolic syndrome, or impaired glucose tolerance. In such populations, portion size, cultivar choice, and concurrent dietary patterns should be carefully considered to avoid potential glycemic excursions.

From the human clinical trials discussed previously, it can be seen that the most frequently occurring positive effect in relation to date consumption is its anti-hypertensive properties. These are, however, influenced by multiple different variables, such as the variety and maturity of the date, the baseline characteristics of patients, and the duration of the study. In these trials, the main varieties of dates used were Ajwa, Medjool, and Hallawi. These specific varieties appeared to be more successful in certain studies that reported reductions in blood pressure among older adult participants [50], positive improvements to blood lipid profiles of overweight or obese patients [8,51], and improvements in blood glucose control in healthy human subjects [7,49]. The studies that observed improvements in these health aspects reported the consumption of dates to be sustained for four to six weeks, as durations of fewer than four weeks did not typically elicit significant benefits in individuals with hypertension or dyslipidemia. As most of these studies lacked demographic diversity and large participant sample sizes, these clinical intervention trials may not be a perfect indicator of the effects that date consumption can exert on hypertensive individuals. Furthermore, though dates have shown positive clinical outcomes, and their bioavailability, safety, and cost effectiveness have been assessed as being acceptable, human clinical studies also indicated a potential lack of optimal dosage and limited investigations of food–drug interactions [8,49].

In conclusion, human clinical trials have shown that dates are effective in preventing or treating multiple risk factors involved in cardiovascular disease, in addition to reducing hypertension, which suggests that dates may be used in preventative measures of the pathogenesis of cardiovascular diseases.

## 5. Conclusions

This review examined whether dates (*Phoenix dactylifera*) can be considered a functional food with relevance for blood pressure regulation and cardiovascular support. Evidence from nutritional, mechanistic, and clinical studies suggests that the combined action of potassium, polyphenols, flavonoids, dietary fiber, and other bioactive constituents may contribute to modest improvements in vascular function and related cardiometabolic parameters.

Preclinical models consistently demonstrate antioxidant, anti-inflammatory, endothelial, and lipid-modulating effects of date-derived compounds, while human studies—though limited in number and scope—report reductions in systolic and diastolic blood pressure, improvements in lipid profiles, and favorable changes in oxidative and inflammatory biomarkers with regular consumption of moderate quantities of dates. These findings, together with the generally favorable safety profile, indicate that dates may be incorporated as part of a supportive dietary strategy for individuals with hypertension.

Nevertheless, current evidence is constrained by variability in date cultivars, processing methods, intervention designs, and dosing protocols. Larger, well-controlled clinical trials across diverse populations are required to clarify efficacy, optimal intake levels, mechanisms of action, and potential interactions with antihypertensive medications. Additional research is also needed to evaluate long-term safety, particularly in populations with diabetes, renal impairment, or other metabolic comorbidities.

In summary, dates show promise as a culturally acceptable and nutritionally rich food that may complement existing dietary approaches for hypertension management. Continued research will be essential to establish standardized recommendations and to determine their role within broader precision nutrition strategies.

## Figures and Tables

**Table 2:** Quantitative outcomes of date consumption in human and animal studies relevant to blood pressure, inflammation, oxidative stress, and lipid metabolism.

Study Type	Model/Population	Date Form and Dose	Duration	Quantitative Outcomes	Reference
Human	Healthy adults (Medjool or Hallawi varieties)	~100 g/day whole dates	4 weeks	↓ Total cholesterol by ~8%; ↓ triglycerides by ~15%; no significant rise in fasting glucose	[45]
Human	Adults with pre-diabetes/Type 2 diabetes	Low-dose date intake (~3 dates/day ≈ 24–30 g)	12 weeks	Improved postprandial glycemic control; ↓ LDL-C by ~6–8%; stable HbA1c	[8]
Human	Older hypertensive participants	~7 Ajwa dates/day (≈ 70–80 g)	6 weeks	↓ SBP by ~14 mmHg; ↓ DBP by ~8.5 mmHg	[47]
Human	Healthy overweight volunteers	100 g/day Hallawi dates	4 weeks	↓ Total cholesterol by ~7%; improved antioxidant status (↓ MDA by ~10–12%)	[7,46]
Animal	Hypercholesterolemic rats	Polyphenol-rich date pulp and seed extract (50–200 mg/kg BW)	4–6 weeks	↓ LDL-C by 20–35%; ↓ CRP and TNF-α significantly; ↑ catalase and GPx activities	[18,33]
Animal	Cardiomyopathy rodent model	Ajwa date extract (300–600 mg/kg BW)	21–30 days	↓ oxidative stress markers (MDA); ↓ IL-6 and TNF-α; improved histological myocardial architecture	[35]
Animal	Hypertensive rats	Date seed extract (various cultivars) at 150–300 mg/kg BW	4–8 weeks	Improved aortic blood flow; ↓ systolic pressure by ~10–20 mmHg; strong ACE-inhibitory activity	[5,39,41]

## Data Availability

No new data were created or analyzed in this study. Data sharing is not applicable to this article.

## Abbreviations

The following abbreviations are used in this manuscript:

ACE	Angiotensin-Converting Enzyme
ACEi	ACE Inhibitor
BMI	Body Mass Index
BHT	Butylated Hydroxytoluene
CRP	C-Reactive Protein
DRI	Dietary Reference Intake
eNOS	Endothelial Nitric Oxide Synthase
GAE	Gallic Acid Equivalent
HDL-C	High-Density Lipoprotein Cholesterol
IL-6	Interleukin-6
LDL	Low-Density Lipoprotein
LDL-C	Low-Density Lipoprotein Cholesterol
MDA	Malondialdehyde
NO	Nitric Oxide
NOx	Nitric Oxide Species
ROS	Reactive Oxygen Species
SOD	Superoxide Dismutase
TNF-α	Tumor Necrosis Factor-Alpha
RE	Retinol Equivalent
CKD	Chronic Kidney Disease
CF	Cardiovascular Function
